# *Sphenomorphus puhoatensis* (Squamata, Scincidae), a new skink from Nghe An Province, Vietnam

**DOI:** 10.3897/zookeys.1279.188050

**Published:** 2026-05-05

**Authors:** Anh Van Pham, Cuong The Pham, An Vinh Ong, Minh Duc Le, Thomas Ziegler, Truong Quang Nguyen

**Affiliations:** 1 Faculty of Environmental Sciences, University of Science, Vietnam National University, Hanoi, 334 Nguyen Trai Road, Hanoi 11416, Vietnam Institute of Zoology, University of Cologne Cologne Germany; 2 Institute of Biology, Vietnam Academy of Science and Technology, 18 Hoang Quoc Viet Road, Hanoi 10072, Vietnam Department of Zoology, Vinh University Vinh Vietnam; 3 Graduate University of Science and Technology, Vietnam Academy of Science and Technology, 18 Hoang Quoc Viet Road, Hanoi 10072, Vietnam Faculty of Environmental Sciences, University of Science, Vietnam National University, Hanoi Hanoi Vietnam; 4 Department of Zoology, Vinh University, 182 Le Duan Road, Nghe An Province, Vinh, Vietnam Central Institute for Natural Resources and Environmental Studies, Vietnam National University Hanoi Vietnam; 5 Central Institute for Natural Resources and Environmental Studies, Vietnam National University, Hanoi, 19 Le Thanh Tong, Hanoi 11021, Vietnam Graduate University of Science and Technology, Vietnam Academy of Science and Technology Hanoi Vietnam; 6 Department of Herpetology, American Museum of Natural History, Central Park West at 79th Street, New York 10024, USA Institute of Biology, Vietnam Academy of Science and Technology Hanoi Vietnam; 7 Cologne Zoo, Riehler Straße 173, 50735, Cologne, Germany Department of Herpetology, American Museum of Natural History New York United States of America; 8 Institute of Zoology, University of Cologne, Zülpicher Straße 47b, 50674, Cologne, Germany Cologne Zoo Cologne Germany

**Keywords:** 16S, COI, forest skink, molecular phylogeny, morphology, new species, Pu Hoat Nature Reserve, *
Sphenomorphus
*, taxonomy

## Abstract

A new species of the genus *Sphenomorphus* Fitzinger, 1843 is described from north-central Vietnam based on morphological differences and molecular divergence. *Sphenomorphus puhoatensis***sp. nov**. can be distinguished from its congeners by distinct body size, head and body scalation, dorsal scale shape, number of lamellae beneath finger IV and toe IV, and color pattern. In the phylogenetic analyses, the new species is recovered as a sister taxon to *S. tamchucensis* and genetically at least 6.9% and up to 16.6% divergent from other species in the genus based on two fragments of the mitochondrial 16S and COI genes, respectively.

## Introduction

The genus *Sphenomorphus* Fitzinger, 1843 currently contains 117 recognized species (Uetz et al. 2026). Among them, 17 species of the genus have been documented from Vietnam (Uetz et al. 2026). Nghe An Province, situated in north-central Vietnam, contains 783,700 hectares of evergreen forest, primarily along the Truong Son Range (The People’s Committee of Nghe An Province 2023). Despite this extensive forest coverage, the province’s biodiversity remains poorly studied, especially with regard to its herpetofauna. Seven new species of amphibians and reptiles have recently been described from Nghe An Province, namely *Leptobrachella puhoatensis* Rowley, Dau & Cao, 2017; *Quasipaa ohlerae* Pham, Nguyen, Hoang, Ziegler, Phan, Pham & Nguyen, 2025; *Amolops tanfuilianae* Sheridan, Phimmachak, Sivongxay & Stuart, 2023; *Kurixalus gracilloides* Nguyen, Duong, Luu & Poyarkov, 2020; *Tylototriton thaiorum* Poyarkov, Nguyen & Arkhipov, 2021; *Ichthyophis griseivermis* Poyarkov, Skorinova, Bragin, Kolchanov, Gorin, Trofimets, Yuzefovich, Le, Nguyen & Skutschas, 2025; and *Achalinus quangi* Pham, Pham, Le, Ngo, Ong, Ziegler & Nguyen, 2023 (Rowley et al. 2017; Nguyen et al. 2020; Poyarkov et al. 2021, 2025; Pham et al. 2023, 2025; Sheridan et al. 2023).

During our recent field work in Nghe An Province, a skink specimen was collected from Pu Hoat Nature Reserve (NR). Morphological and molecular phylogenetic analyses revealed that the newly collected specimen represents an unnamed species of *Sphenomorphus*. Thus, we herein describe the skink from Nghe An Province, Vietnam, as a new species.

## Materials and methods

### Sampling

**Figure 1.**
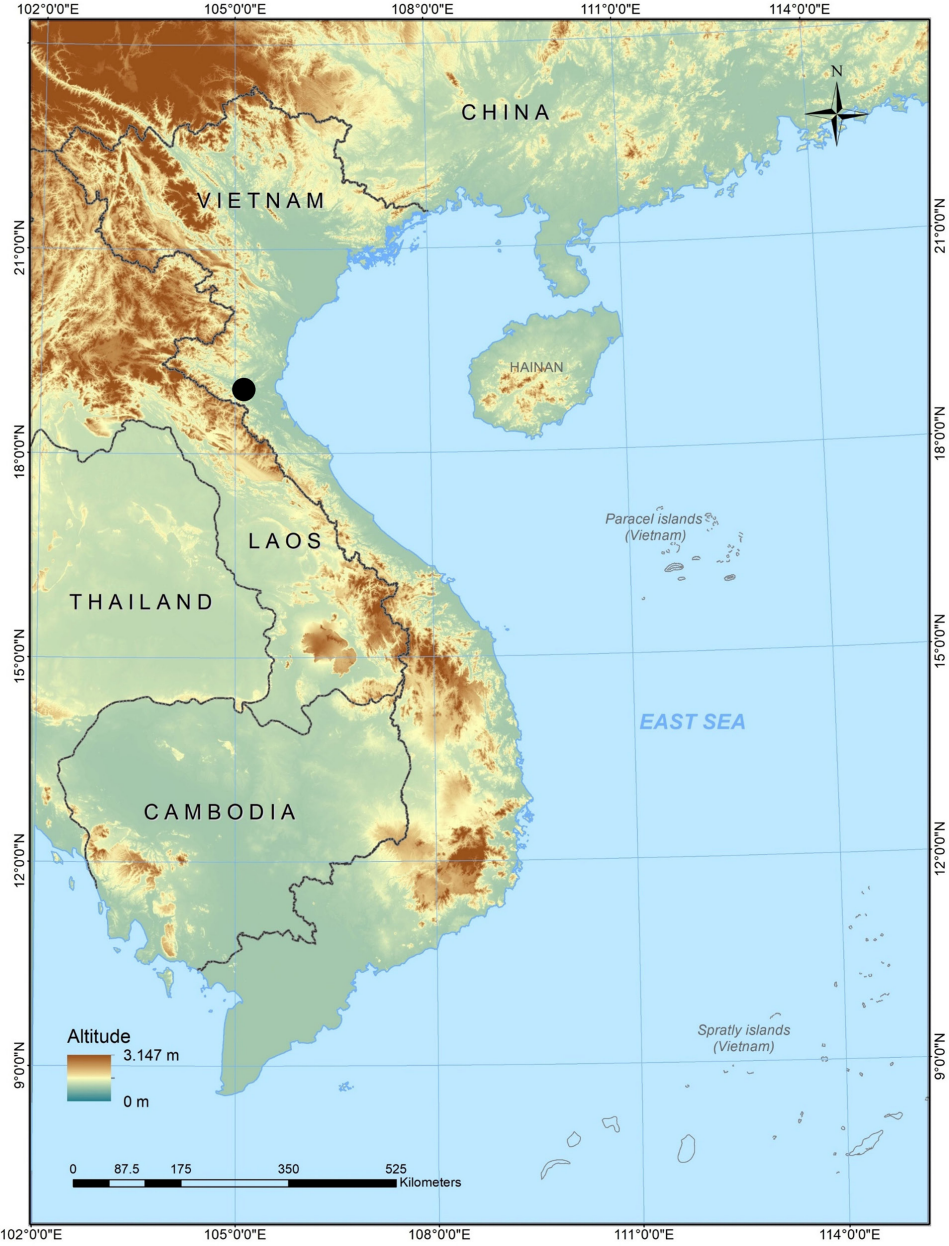
Map showing the type locality (black circle) of *Sphenomorphus puhoatensis* sp. nov. in Nghe An Province, Vietnam.

A field survey was conducted in June 2025 in the evergreen forest of Pu Hoat NR, Nghe An Province, north-central Vietnam (Fig. 1). After being photographed in life, the skink was anesthetized and euthanized in a closed vessel with a piece of cotton wool containing ethyl acetate (Simmons 2002), fixed in 85% ethanol for ten hours, and then transferred to 75% ethanol for permanent storage. Tissue samples were preserved separately in 70% ethanol before fixation. A voucher specimen was deposited in the Institute of Biology (**IB**), Vietnam Academy of Science and Technology, Hanoi, Vietnam.

### Molecular data and phylogenetic analyses

We sequenced one sample of the newly collected skink specimen from Nghe An Province. Additionally, 20 ingroup and two outgroup taxa were included in the phylogenetic analyses following Nguyen et al. (2018) and Pham et al. (2026) (Table 1). The tissue sample was extracted using a DNeasy blood and tissue kit (Qiagen, Hilden, Germany). Extracted DNA from fresh tissue was amplified using a DreamTaq PCR mastermix (Thermo Fisher Scientific, Lithuania). A fragment of the mitochondrial cytochrome *c* oxidase subunit I (COI) was sequenced using the primer pair LCO1490 (5’-GGT CAA CAA ATC ATA AAG ATA TTG G- 3’) and HCO2198 (5’-TAA ACT TCA GGG TGA CCA AAA AAT CA-3’) (Jeong et al. 2013) and the primer pair L2606 (5’-CTGACCGTGCAAAGGTAGCGTAATCACT- 3’; forward), H3056 (5’-CTCCGGTCTGAACTCAGATCACGTAGG-3’; reverse) (Hedges et al. 1993) was used to amplify a fragment of the mitochondrial 16S rRNA gene. The PCR reaction volume was 21 μl (10 μl of mastermix, 5 μl of water, 2 μl of each primer at 10 pmol/μl, and 2 μl of DNA or higher depending on the quantity of DNA in the final extraction solution). The PCR conditions for both COI and 16S were 95 °C for 5 min to activate the taq; with 40 cycles at 95 °C for 30 s, 50 °C for 45 s, 72 °C for 60 s; and a final extension at 72 °C for 6 min.

**Table 1.** Samples of 16S and COI genes used in this study.

Voucher	Species	Locality	GenBank accession number (16S)	GenBank accession number (COI)	References
**IB R.6466**	*** Sphenomorphus puhoatensis* sp. nov**.	**Nghe An, Vietnam**	**PZ245459**	**PZ245458**	**This study**
FMNH 267739	* S. annamiticus*	Koh Kong Province, Cambodia	KX398012		Grismer et al. (2019)
IEBR 39484	* S. annamiticus*	Gia Lai, Vietnam	HM773221		Grismer et al. (2019)
IEBR 39474	* S. buenloicus*	Gia Lai, Vietnam	HM773218		Nguyen et al. (2011)
CIB 119027	* S. cryptotis*	Sichuan, China	OP942190	OP942215	Jia et al. (2024)
EMD 368	* S. diwata*	Mindanao, Philippines	JF498085		Linkem et al. (2011)
EMD 428	* S. diwata*	Mindanao, Philippines	JF498086		Linkem et al. (2011)
FBRC_DNA207	* S. dussumieri*	Ghats, India		MK089440	Ganesh et al. (2018)
KU 315061	* S. fasciatus*	Mindanao, Philippines	JF498088		Linkem et al. (2011)
KUZ 37239	* S. indicus*	Yunlin, Taiwan, China	AB028820		Sumarli et al. (2016)
HS17088	* S. indicus*	Tunxi, Huangshan, Anhui, China	MK450438	MK450438	Tang et al. (2019)
NC41124	* S. incognitus*	Huangshan, Anhui, China	NC41124	NC41124	Tang et al. (2019)
MH329292	* S. incognitus*	Huangshan, Anhui, China	MH329292	MH329292	Tang et al. (2019)
ZMMU R-13680-00094	* S. maculatus*	Dong Nai, Vietnam		MH119629	Neang et al. (2018)
USNM: Herp: 587039	* S. maculatus*	Tanintharyi, Myanmar		MG935700	Mulcahy et al. (2022)
KUZ 37239	* S. maculatus*	Kaeng Krachan, Thailand	AB028821		Sumarli et al. (2016)
E6113	* S. maculatus*	Thailand	AY308308		Honda et al. (2003)
KUZ 21251	* S. praesignis*	Bukit Larut, Malaysia	AB028822		Sumarli et al. (2016)
CAS 236398	* S. scutatus*	Malakal Island, Palau	JF498093		Linkem et al. (2011)
USNM:Herp:585412	* S. scutatus*	Ngermalk Island, Palau		MH274672	Mulcahy et al. (2022)
USNM:Herp:533601	* S. solomonis*	Temotu, Solomon Islands		MH274678	Mulcahy et al. (2022)
USNM:Herp:533597	* S. solomonis*	Temotu, Solomon Islands		MH274679	Mulcahy et al. (2022)
LSUHC 11722	* S. sungaicolus*	Hutan Lipur Sekayu, Terengganu, Malaysia	KX398013		Sumarli et al. (2016)
LSUHC 11780	* S. sungaicolus*	Hutan Lipur Sekayu, Terengganu, Malaysia	KX398014		Sumarli et al. (2016)
LSUHC 9041	* S. tersus*	Perlis, Malaysia	KX398015		Sumarli et al. (2016)
USNM:Herp:595507	* S. tonkinensis*	Hai Phong, Vietnam	OM387054	OM420325	Miller et al. (Unpublished)
USNM:Herp:595508	* S. tonkinensis*	Hai Phong, Vietnam	OM387055	OM420326	Miller et al. (Unpublished)
IB R.6455	* S. tamchucensis*	Ninh Binh, Vietnam	PZ245460	PX664538	Pham et al. (2026)
IB R.6458	* S. tamchucensis*	Ninh Binh, Vietnam	PZ245461	PX664543	Pham et al. (2026)
KU 309900	* S. variegatus*	The Philippines	JF498096		Sumarli et al. (2016)
KU 315087	* S. variegatus*	The Philippines	JF498096		Sumarli et al. (2016)
ZMMU_NAP-07141	* S. veunsaiensis*	Gia Lai, Vietnam		PP931018	Bragin et al. (2025)
ZMMU_NAP-05515	* S. veunsaiensis*	Gia Lai, Vietnam		PP931016	Bragin et al. (2025)
ITBCZ 5684	* S. yersini*	Khanh Hoa, Vietnam		MH237971	Nguyen et al. (2018)
ITBCZ 5685	* S. yersini*	Khanh Hoa, Vietnam		MH237972	Nguyen et al. (2018)
USNM:Herp:595505	* Sphenomorphus * sp.	Tuyen Quang, Vietnam		OM420415	Miller et al. (Unpublished)
	* Plestiodon elegans*	China	KJ643142		Song et al. (2016)

PCR products were subjected to electrophoresis using a 1% agarose gel at 1^st^ BASE (Selangor, Malaysia). Gels were stained for 10 min in 1X TBE buffer at 2 pg/ml of ethidium-bromide and visualized under UV light. Successful amplifications were purified to eliminate PCR components using a GeneJET™ PCR Purification Kit (Thermo Fisher Scientific, Lithuania). Clean PCR products were sent to 1^st^ BASE (Malaysia) for sequencing. Sequences generated in this study were edited using Geneious v. 7.1.8 (Kearse et al. 2012).

After sequences were aligned using Clustal X v. 2 (Thompson et al. 1997), data were analyzed using maximum parsimony (MP), as implemented in PAUP* v. 4.0a169 (Swofford 2001), and Bayesian inference (BI), as implemented in MrBayes v. 3.2.7 (Ronquist et al. 2012). For MP analysis, heuristic analysis was conducted with 100 random taxon addition replicates using the tree-bisection and reconnection (TBR) branch-swapping algorithm, with no upper limit set for the maximum number of trees saved. Bootstrap support was calculated using 1000 pseudo-replicates and 100 random taxon addition replicates. All characters were equally weighted and unordered. For the maximum likelihood (ML) analysis, we used IQ-TREE v. 2.3.4 (Nguyen et al. 2015) with a single model and 10,000 ultrafast bootstrap replications. The optimal model for nucleotide evolution was determined using jModeltest v. 2.1.4 (Darriba et al. 2012).

For Bayesian analyses, we used the optimal model selected by jModeltest with parameters estimated using MrBayes v. 3.2.7. Two independent analyses with four Markov chains (one cold and three heated) were run simultaneously for ten million generations with a random starting tree and sampled every 1000 generations. Log-likelihood scores of sample points were plotted against generation time to determine stationarity of Markov chains. Trees generated before log-likelihood scores reached stationarity were discarded from the final analyses using the burn-in function. The posterior probability values for all clades in the final majority rule consensus tree were provided. The optimal model for nucleotide evolution was set to GTR+I+G for ML and single-model Bayesian analyses as selected by jModeltest v. 2.1.4. The cutoff point for the burn-in function was set to 25% of the generated trees. Nodal support was also evaluated using bootstrap replication (BP) as estimated in PAUP, ultrafast bootstrap (UFB) in IQ-TREE v. 2.3.4, and posterior probabilities (PP) in MrBayes v. 3.2.7. BP ≥ 70, PP ≥ 0.95, and UFB ≥ 95 were regarded as strong clade support (Hillis and Bull 1993; Ronquist et al. 2012; Nguyen et al. 2015). Uncorrected pairwise divergences were calculated in PAUP* v. 4.0b10.

### Morphological examination

Measurements were taken with a digital caliper (Electronic Digital Caliper) to the nearest 0.1 mm. The following morphological characteristics were recorded (after Nguyen et al. 2011):

**SVL** snout-vent length (from tip of snout to cloaca);

**TaL** tail length (from cloaca to tip of tail);

**AG** distance from posterior junction of forelimb and body wall to anterior junction of hindlimb and body wall (with the limbs held at right angles to the body);

**HL** head length (from tip of snout to posterior margin of parietal or interparietal, depending on the longest distance);

**HW** head width (at the widest portion of temporal region);

**HH** head height (at the deepest portion of temporal region);

**SL** snout length (from anterior margin of eye to tip of snout);

**STL** distance from snout to anterior border of tympanum;

**SFlL** snout-forelimb length (from tip of snout to anterior junction of forelimb and body wall, with the limb held at right angles to the body);

**END** distance from anterior margin of eye to posterior border of nostril;

**EL** eye length (distance between anterior and posterior corners of eyelid);

**TYD** maximum diameter of tympanum;

**FlL** forelimb length (from anterior junction of forelimb and body wall to the tip of fourth finger, with the limb held at right angles to the body);

**HlL** hindlimb length (from anterior junction of hindlimb and body wall to the tip of fourth toe, with the limb held at right angles to the body).

### Scalation

NSB: number of scales bordering posterolateral border of parietal; So: supraoculars; Nu: nuchals; Lo: loreals; PC: preoculars; PS: presuboculars; SC: supraciliaries; Po: postoculars; Pos: postsuboculars; PT: primary temporals; ST: secondary temporals; S: supralabials; AL: auricular lobules; IF: infralabials; CS: chin shields (pairs); MB: midbody scale rows; PV: paravertebral scale rows; VR: ventral scale rows; PR: precloacals; FL4: subdigital lamellae on fourth finger; TL4: subdigital lamellae on fourth toe. Bilateral scale counts were given as left/right.

### Statistical analyses

Although only a single specimen was available, statistical analyses were conducted to compare the newly discovered population with its closest relatives based on the phylogenetic tree of *Sphenomorphus*. Raw morphological data for these analyses were obtained from the new specimen collected in Pu Hoat NR, as well as from 34 specimens representing four other *Sphenomorphus* species documented in previous studies (Nguyen 2011).

All statistical analyses were conducted in GroupStruct2 by R v. 4.5.2 R Core Team 2025) (see Chan and Grismer 2025). To remove the effects of allometry in the morphometric characters, morphometric data were also normalized to adjust raw data of morphometrics using the following equation: Xadj = log(X) – ß[log(SVL) − log(SVLmean)], where Xadj = adjusted value; X = measured value; ß = unstandardized regression coefficient for each sample and SVLmean = overall average SVL of all samples (Thorpe 1975, 1983; Turan 1999; Lleonart et al. 2000; Chan and Grismer 2022, 2025).

Morphospatial relationships among species were visualized using principal component analysis (PCA) based on 13 size-corrected morphometric characters (SVL, AG, SL, STL, SFlL, END, EL, HL, HW, HH, TYD, FlL, HlL) and 12 meristic characters (So, Nu, PC, PS, SC, PT, S, IF, MB, PV, VR, FL4, TL4) to compare differences in body shapes and recover characters contributing to those differences. A multiple factor analysis (MFA, Grismer 2025) was conducted to assess morphospace between the skink from Pu Hoat NR and its close relatives using the GroupStruct2 (Grismer 2025).

## Results

### Phylogenetic analyses

**Figure 2.**
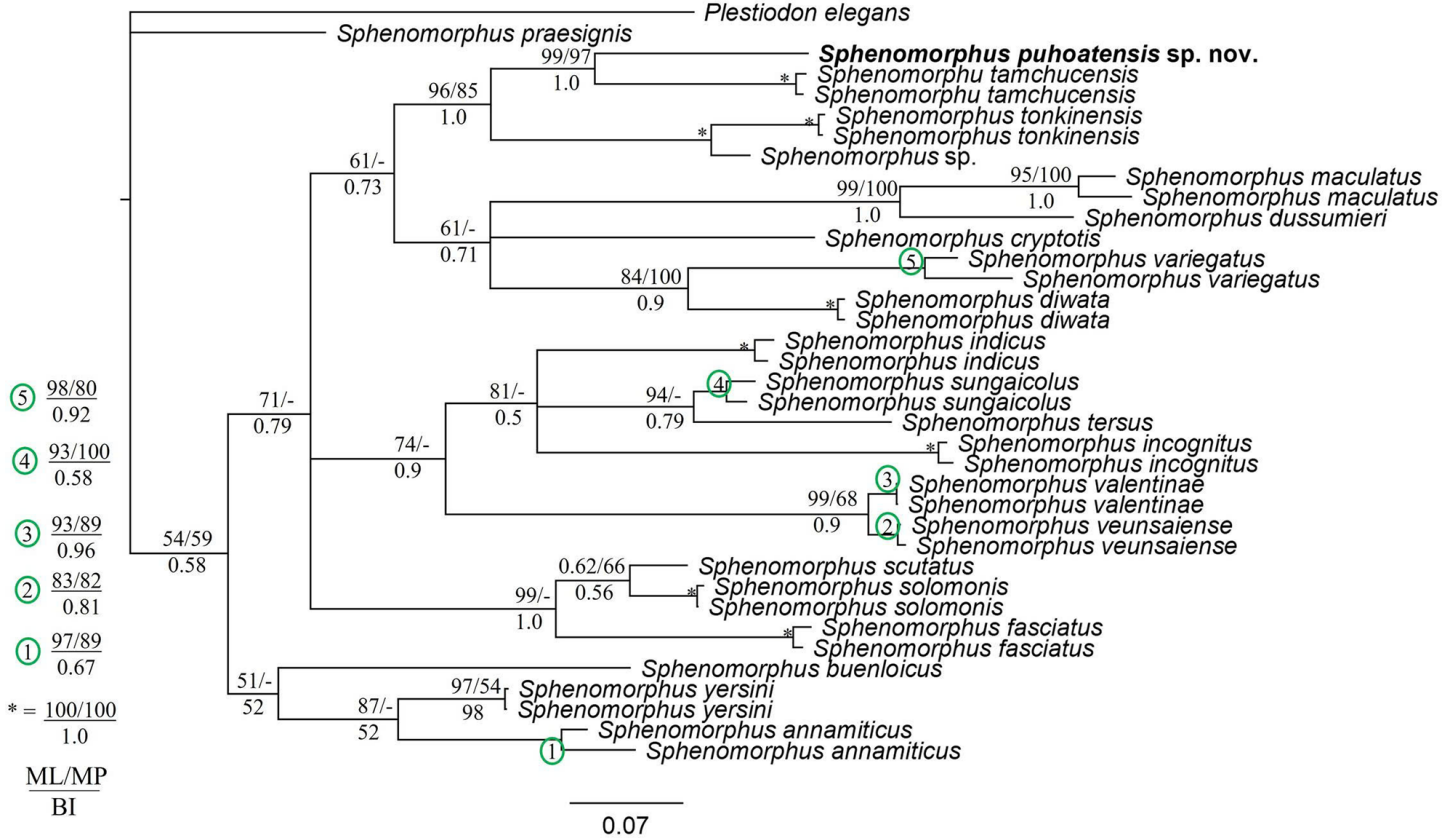
Bayesian phylogeny based on 16S and COI genes. Numbers above and below branches are ML ultrafast bootstrap/MP bootstrap values and single-model Bayesian posterior probabilities (> 50%), respectively. Dash indicates unsupported node.

The combined matrix of the 16S rRNA and COI contained 1138 aligned characters (16S: 488 and COI: 650), of which 732 were constant, 380 parsimony-informative and 26 variable character parsimony-uninformative. The MP analysis produced a single most parsimonious tree (tree length = 1648, consistency index = 0.43, retention index = 0.64). The new species from Nghe An Province, Vietnam was strongly supported as a member of the clade consisting of *S. tamchucensis* Pham, Pham, Ha, Le, Phan, Ho, Le & Nguyen, 2026, *S. tonkinensis* Nguyen, Schmitz, Nguyen, Orlov, Böhme & Ziegler, 2011, and *Sphenomorphus* sp. (UFB = 96, BP = 85, PP = 1.0) (Fig. 2). It was also recovered as a sister taxon to *S. tamchucensis* with significant support from all analyses (UFB/BP/PP = 99/97/1.0). In terms of genetic divergence, the new species is most closely related to *S. tamchucensis* and the two species are separated by 16.6–17.2% in COI and 6.9–7.1 in 16S (Suppl. material 1: tables S1, S2).

### Statistical analyses

**Figure 3.**
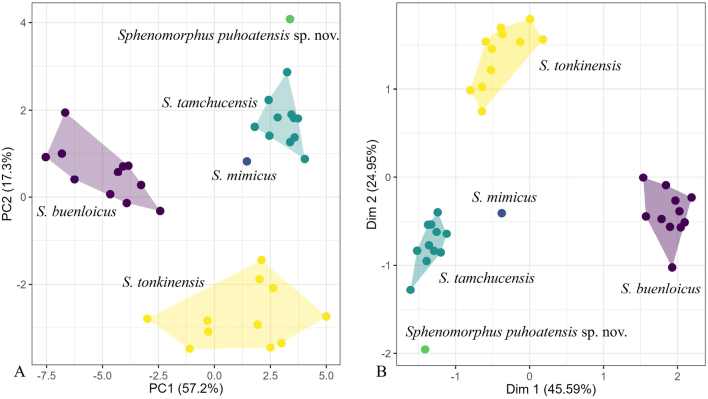
PCA (**A**) and MFA (**B**) scatter plot showing the morphospatial relationships among the *Sphenomorphus puhoatensis* sp. nov. and its closely related species.

The PCA results indicated that the body shape of the skink specimen from Nghe An Province and *S. tamchucensis* from Ninh Binh Province are more similar to each other than to *S. buenloicus* Darevsky & Nguyen, 1983, *S. mimicus* Taylor, 1962, and *S. tonkinensis* (Fig. 3). The clustering of species in the PCA showed the first two principal components (PC1 and PC2) recovered 74.5% of the variation in the normalized morphometric and meristic data set (Fig. 3). PC1 accounted for 57.2% of the variation in the data set and loaded most heavily for HL, SL, SFIL, STL, HW, FIL, HIL, EL, and SVL. PC2 accounted for an additional 17.3% of the data set and loaded most heavily for PS, PV, VR, MB, FL4, and S (Suppl. material 2). The PCA revealed that the newly discovered species is clearly separated from most other species and, more importantly, distinctly divergent from its closest relatives (Fig. 3).

**Figure 4.**
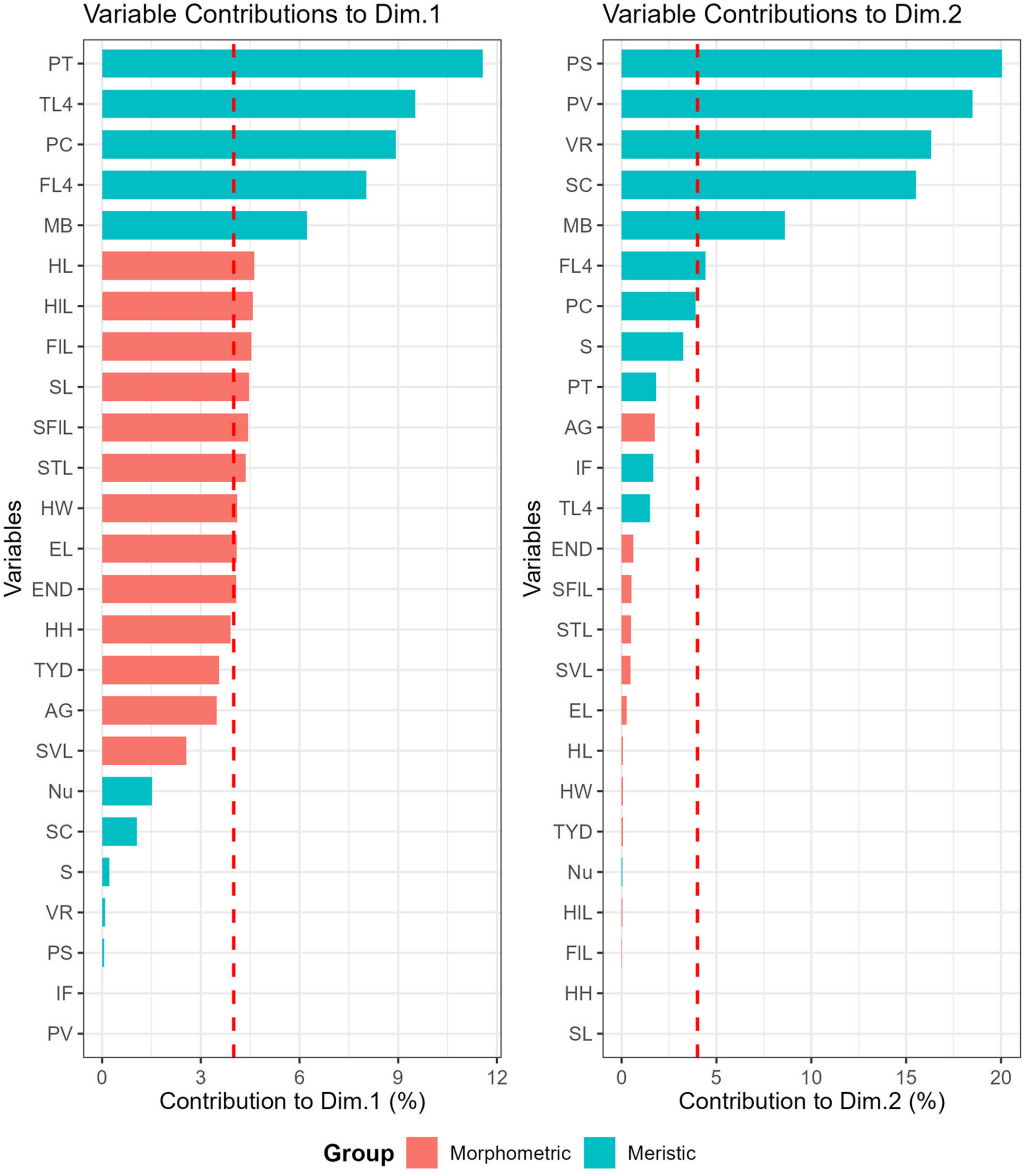
Trait Contributions to Dimensions 1 and 2 of the MFA.

The MFA largely mirrored the PCA, showing that the specimen from Nghe An Province is closely aligned along Dimension 1 (Dim1) and distinctly separated from *S. tamchucensis*, whereas *S. buenloicus*, *S. mimicus*, and *S. tonkinensis* occupied intermediate positions. Dim1 accounted for only 45.59% and Dim2 24.95% of the variation in the total evidence data set (Fig. 3). Dim1 is loaded most heavily for PT, TL4, PC, FL4, and MB, while Dim2 is loaded most heavily for PS, PV, VR, SC, FL4, PC, S, and PT (Fig. 4).

### Taxonomic account

#### Sphenomorphus puhoatensis sp. nov.

Taxon classificationAnimaliaSquamataScincidae

EF1BEF71-871B-51E3-B709-80AC77BF33F2

https://zoobank.org/9802111D-C78D-4E44-8FD8-EF15FCC96B6B

Figs 5, 6, Table 2

##### Material examined.

***Holotype***. • ♂ IEBR R.6466 (Field number PH NA 2025.86), collected on 28 June 2025 by A.V. Ong, T.Q. Phan, and N.H. Nguyen in the evergreen forest (19°71'347"N, 104°83'748"E, at an elevation of 1071 m a.s.l.), within Pu Hoat Nature Reserve, Nghe An Province, Vietnam.

##### Diagnosis.

The new species can be distinguished from other species of *Sphenomorphus* by a combination of the following morphological characteristics: size medium (SVL 38.5 mm); primary temporals two; external ear openings present, without lobules; loreals two; supralabials six; infralabials six; enlarged nuchals in one pair; midbody scales in 28 rows; dorsal scales smooth, in six rows across the back; paravertebral scales 61, not widened; ventral scales in 56 rows; seven smooth lamellae beneath finger IV and 12 or 13 beneath toe IV; toes not reaching to fingers when limbs adpressed along body; dorsal surface of body and tail bronze brown with a discontinuous dark vertebral stripe, from middle of neck to tail base; a black stripe, two scales wide, running from nostril to eye and extending from posterior margin of eye along upper part of flank and tail middle.

##### Description of holotype.

Size medium (SVL 38.5 mm), tail tip lost (TaL 48.4 mm); head longer than wide (HL 6.6 mm, HW 4.9 mm); for further measurements see Table 2; snout obtuse, round anteriorly; rostral wider than high, distinctly visible from above; supranasals absent; frontonasal wider than long, in contact with rostral, nasals, postnasals, and prefrontals; prefrontals not in contact with each other; frontal narrowing posteriorly, about 1.3 times longer than the distance to the tip of snout, in contact with prefrontals, first to third supraoculars, and frontoparietals; frontoparietals in contact with each other, and bordered by frontal, three posterior supraoculars, parietals, and interparietal; interparietal lozenge-shaped with a small transparent spot in posterior angle; parietals in contact posteriorly, posterolateral border surrounded by seven scales; enlarged nuchals in one pair; nostril in center of nasal; postnasal single; loreals two, posterior loreal in contact with anterior loreal, anterior supraciliary, preocular, and second supralabial; preocular single; presuboculars 1/3, posterior scale in contact with second and third supralabials; supraciliaries seven, first largest, first to third in contact with first supraocular, posterior portion of third to fourth in contact with second supraocular, fifth to sixth in contact with third supraocular, posterior portion of sixth to seventh in contact with fourth supraocular; supraoculars four, third widest, fourth supraocular followed by two small scales; postoculars two; postsuboculars three; primary temporals two, lower one in contact with fifth and sixth supralabials, upper one very small; secondary temporals two, upper one very large, overlapped by lower one, in contact with parietal; lower eyelid scaly, separated from supralabials by a row of small scales; supralabials six, fifth enlarged, fourth below the eye; infralabials six, first very small; external ear openings nearly round, smaller than eye length, without lobules; tympanum sunk; mental wider than long, round anteriorly, in contact with anterior infralabial on each side and postmental; postmental undivided, in contact with mental, first and second infralabials, and anterior pair of chin shields; three pairs of chin shields, anterior pair in contact with each other anteriorly, second pair separated from each other by a gular scale, and posterior pair separated from each other by three scales; midbody scales in 28 rows; dorsal scales smooth, subequal to lateral and ventral scales, 1/2 + 6 + 1/2 scale rows between dark stripes on upper lateral zones; paravertebral scales 61, not widened; ventrals smooth, in 56 rows; precloacals four, inner scales overlapping outer ones, medial two enlarged, right scale overlapped by left scale; tai thick at base, median subcaudals on posterior part of tail slightly widened. Limbs well developed, pentadactyl; third and fourth fingers equal in length; subdigital lamellae smooth, 7/7 under fourth finger and 13/12 under fourth toe; toe and finger separated when adpressed along body, adpressed forelimb reaching to the position between tympanum and eye.

**Table 2.** Morphological characteristics of the holotype (IB R.6466, adult male) of *Sphenomorphus puhoatensis* sp. nov. from Nghe An Province, Vietnam.

Measurements (in mm)		Scalation	
SVL	38.5	Supraoculars	4
TaL	48.4	Nuchals	1/1
AG	22.2	Loreals	2
SL	2.7	Preocular	1
STL	7.5	Presubocular	1
SFlL	13.2	Supraciliaries	7/7
END	1.4	Postoculars	4/3
EL	2.2	Postsuboculars	2
HL	6.6	Primary temporals	2
HW	4.9	Secondary temporals	2
HH	3.2	Supralabials	6/6
TYD	0.9	Auricular lobules	absent
FlL	8.0	Infralabials	6/6
HlL	13.2	Chin shields (pairs)	3
FIL/SVL	0.21	Midbody scale rows	28
HIL/SVL	0.34	Dorsal scale rows between dark stripes on upper lateral zones	1/2 + 6 + 1/2
AG/SVL	0.58	Paravertebral scales	61
STL/SVL	0.19	Ventrals in transverse rows	56
HL/SVL	0.17	Precloacals (enlarged)	2
HW/SVL	0.13	Lamellae on finger IV	7/7
HH/SVL	0.08	Lamellae on toe IV	13/12

##### Coloration in preservative.

**Figure 5.**
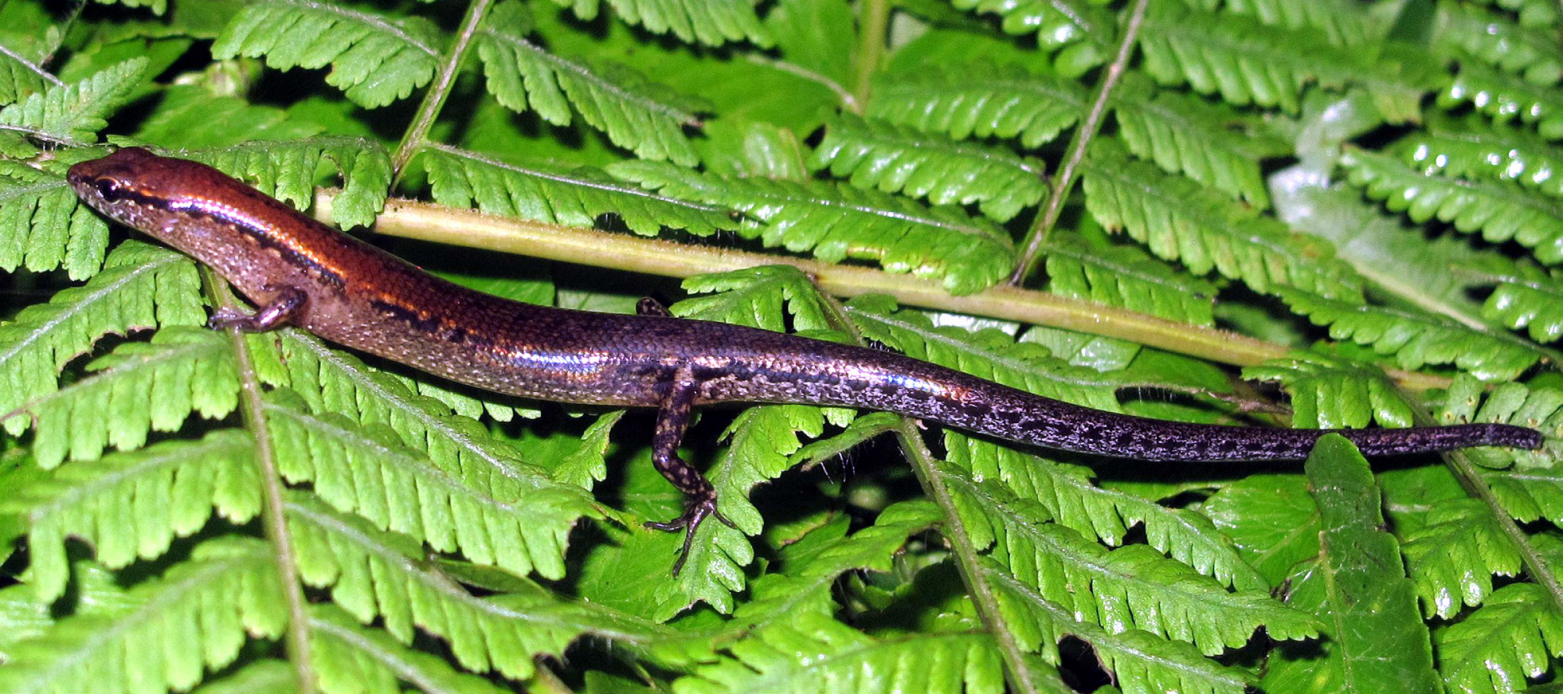
Holotype of *Sphenomorphus puhoatensis* sp. nov. (IB R.6466, adult male) in life.

Dorsal surface of body and tail bronze brown with a discontinuous dark vertebral stripe, from middle of neck to tail base; a black stripe, two scales wide, running from nostril to eye and extending from posterior margin of eye along upper part of flank and tail middle, interrupted by small light spots from behind the neck, paler in posterior part of tail; supralabials and infralabials with dark bars on sutures; lateral side of the head and flank light brown, with dark spots; upper of limbs brown with light spots; chin, throat, venter, tail base, underside of fore and hind limbs cream; underside of tail with very small grey-brown dots. For coloration in life see Fig. 5.

**Figure 6.**
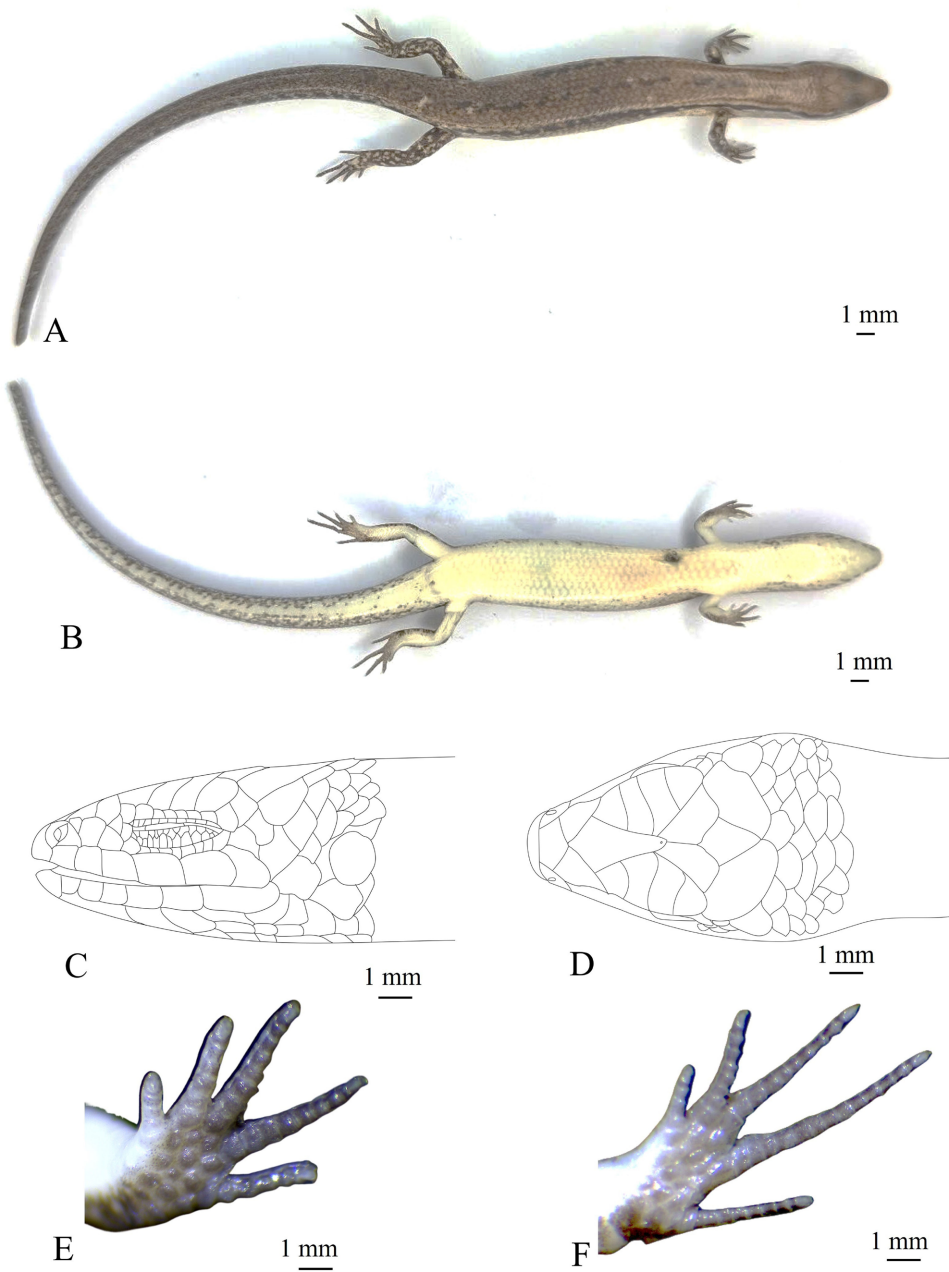
Holotype of *Sphenomorphus puhoatensis* sp. nov. (IB R.6466) in preservative. **A**. Dorsal view; **B**. Ventral view; **C**. Lateral view; **D**. Dorsal view of head; **E**. Ventral view of hand; **F**. Ventral view of foot.

##### Distribution.

*Sphenomorphus puhoatensis* sp. nov. is currently known only from the type locality in Nghe An Province, Vietnam (Fig. 1).

##### Natural history.

**Figure 7.**
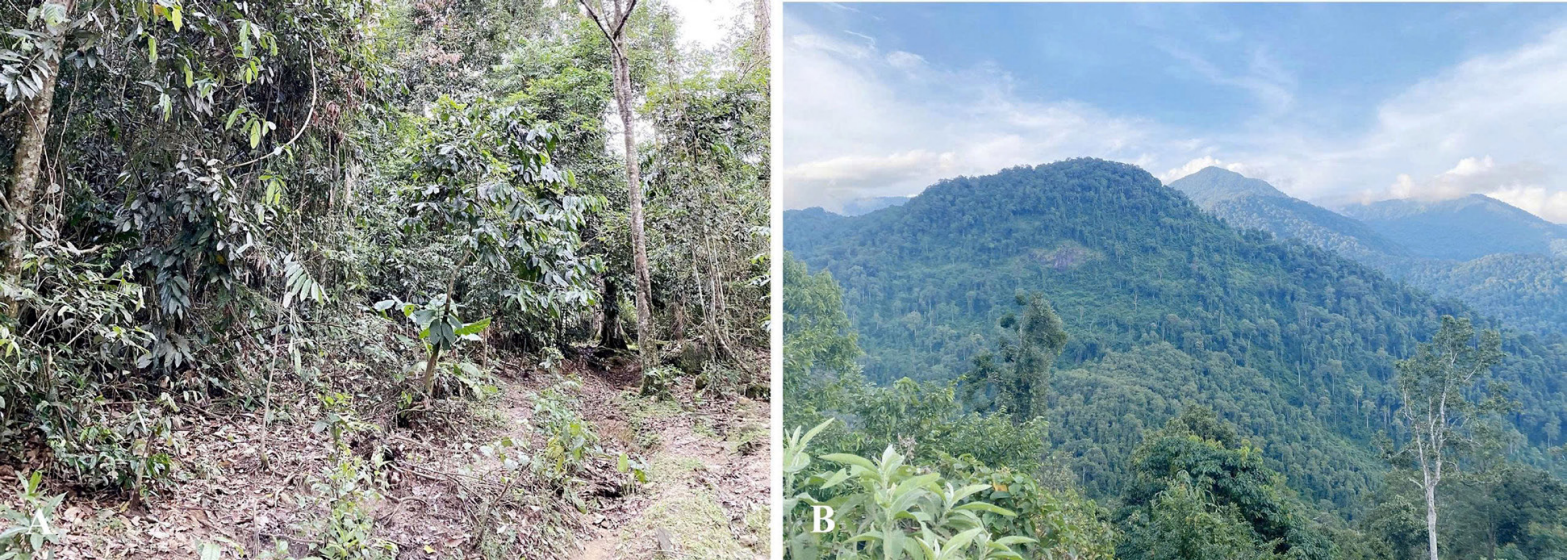
Habitat of *Sphenomorphus puhoatensis* sp. nov. in Pu Hoat Nature Reserve, Nghe An Province, Vietnam.

The type specimen was collected at 21:00, on the ground, in a forest path. The surrounding habitat was evergreen forest composed of large, medium and small hardwoods mixed with shrubs (Fig. 7). Air temperatures ranged from 23.4–28.6 °C and relative humidity was 85–93% at the sites. Other reptile species encountered at the sites included *Physignathus cocincinus* Cuvier; *Hebius* sp.; *Pareas hamptoni* (Boulenger); *Pareas margaritophorus* (Jan); and *Trimeresurus cf. stejnegeri* Schmidt.

##### Etymology.

The specific name “*puhoatensis*” is derived from Pu Hoat Nature Reserve, the type locality of the new species in Nghe An Province, Vietnam. For the common names, we suggest Pu Hoat Forest Skink (English) and Thằn lằn phê-nô pù hoạt (Vietnamese).

##### Comparisons.

We compared the new species with other known taxa in the genus *Sphenomorphus* from Vietnam, Laos, Myanmar, southern China, Cambodia, Thailand, and Peninsular Malaysia. Morphological comparisons were based on data from the following literature: Duméril and Bibron (1839), Boulenger (1890), Van Denburgh (1912), Smith (1924, 1935), Taylor (1963), Eremchenko (2003), Nguyen et al. (2010, 2011, 2013, 2018), Grismer and Quah (2015), Sumarli et al. (2016), Grismer et al. (2019, 2020), Le et al. (2020), Bragin et al. (2025), and Pham et al. (2026).

*Sphenomorphus puhoatensis* sp. nov. differs from *S. annamiticus* (Boettger) by having more midbody scale rows (28 vs 24), fewer lamellae under toe IV (12 or 13 vs 17–19), and fewer lamellae under finger IV (7 vs 11–14); from *S. anomalopus* (Boulenger) by having fewer midbody scale rows (28 vs 38) and a smaller size (SVL 38.5 mm vs 70 mm); from *S. bacboensis* (Eremchenko) by having fewer midbody scale rows (28 vs 30–32), fewer lamellae under toe IV (12 or 13 vs 14–17), and more supraciliaries (8 vs 6); from *S. buenloicus* by having fewer midbody scale rows (28 vs 32–34), fewer lamellae under toe IV (12 or 13 vs 16–19), more anterior temporals (2 vs 1), and a smaller size (SVL 38.5 mm vs 56 mm); from *S. cameronicus* Smith by having a smaller size (SVL 38.5 mm vs 70 mm) and fewer midbody scale rows (28 vs 38); from *S. cryptotis* Darevsky, Orlov & Ho by having a smaller size (SVL 38.5 mm vs 58–79 mm), fewer midbody scale rows (28 vs 32–38), fewer lamellae under toe IV (12 or 13 vs 17–23), and fewer paravertebral scale rows (61 vs 71–89); from *S. grandisonae* Taylor by having a larger size (SVL 38.5 mm vs 30.0 mm), fewer midbody scale rows (28 vs 34), and the presence of enlarged nuchals (vs absent); from *S. helenae* Cochran by having fewer midbody scale rows (28 vs 30) and the presence of an interrupted lateral stripe (vs continuous); from *S. incognitus* (Thompson) by having a smaller size (SVL 38.5 mm vs 80–103 mm), fewer midbody scale rows (28 vs 36–40), fewer paravertebral scale rows (61 vs 67–80), and fewer lamellae under toe IV (12 or 13 vs 19–24); from *S. indicus* (Gray) by having a smaller size (SVL 38.5 mm vs 61–90 mm), fewer paravertebral scale rows (61 vs 65–77), and fewer lamellae under toe IV (12 or 13 vs 16–20); from *S. lineopunctulatus* Taylor by having a smaller size (SVL 38.5 mm vs 84 mm), fewer midbody scale rows (28 vs 38), and fewer paravertebral scale rows (61 vs 76); from *S. maculatus* (Blyth) by having a smaller size (SVL 38.5 mm vs 62 mm), fewer midbody scale rows (28 vs 38–42), fewer paravertebral scale rows (61 vs 69–78), and fewer lamellae under toe IV (12 or 13 vs 18–21); from *S. malayanus* (Doria) by having fewer ventral scales (56 vs 74) and fewer paravertebral scales (61 vs 76–80); from *S. mimicus* by having fewer midbody scale rows (28 vs 30), fewer supraciliaries (8 vs 9), and few lamellae under fourth toe (12 or 13 vs 16); from *S. orientale* (Shreve) by having more midbody scale rows (28 vs 24–26) and fewer ventral scales (56 vs 69–71); from *S. praesignis* (Boulenger) by having a smaller size (SVL 38.5 mm vs 109 mm) and fewer lamellae under finger IV (7 vs 12–15); from *S. phuquocensis* Grismer, Nazarov, Bobrov & Poyarkov by having a smaller size (SVL 38.5 mm vs 60 mm), more midbody scale rows (28 vs 23), fewer lamellae under finger IV (7 vs 14), and fewer lamellae under toe IV (12 or 13 vs 18 or 19); from *S. preylangensis* Grismer, Wood, Quah, Anuar, Poyarkov, Thy, Orlov, Thammachoti & Seiha by having a smaller size (SVL 38.5 mm vs 51.4–87.6 mm), more midbody scale rows (28 vs 24), fewer lamellae under finger IV (7 vs 11–14), and fewer lamellae under toe IV (12 or 13 vs 17–19); from *S. sanctus* (Duméril & Bibron) by having a fewer paravertebral scales (61 vs 71), fewer supraoculars (4 vs 5), and fewer lamellae under toe IV (12 or 13 vs 26–27); from *S. scotophilus* (Boulenger) by having fewer supraoculars (4 vs 5) and fewer lamellae under fourth toe (12 or 13 vs 22–23); from *S. senja* Grismer & Quah by having a smaller size (SVL 38.5 mm vs 60–65 mm), fewer paravertebral scales (61 vs 72–73), and fewer ventral scale rows (60–67 vs 68); from *S. shelfordi* (Boulenger) by having a smaller size (SVL 38.5 mm vs 67 mm) and fewer lamellae under toe IV (12 or 13 vs 28–29); from *S. stellatus* (Boulenger) by having a smaller size (SVL 38.5 mm vs 80 mm), more midbody scale rows (28 vs 24) and the absence of two enlarged vertebral scale rows (vs present); from *S. sungaicolus* Sumarli, Grismer, Wood, Ahmad, Rizal, Ismail, Izam, Ahmad & Linkem by having a smaller size (SVL 38.5 mm vs 67–90 mm), fewer midbody scale rows (28 vs 39–44), fewer paravertebral scales (61 vs 72–81), and fewer ventral scale rows (56 vs 74–86); from *S. tersus* (Smith) by having a smaller size (SVL 38.5 mm vs 90–92 mm) and two loreals (vs three); from *S. tetradactylus* (Darevsky & Orlov) by having more midbody scale rows (28 vs 20), more paravertebral scales (61 vs 47), more ventral scales (56 vs 50), more lamellae under fourth toe (12 or 13 vs 10), forelimb pentadactyl (vs tetradactyl), and the presence of external ear openings (vs absent); from *S. tridigitus* (Bourret) by having more midbody scale rows (28 vs 18–20), more paravertebral scales (61 vs 52), more ventral scales (56 vs 52), more lamellae under fourth toe (12 or 13 vs 7 or 8), forelimb pentadactyl (vs tridactyl), and the presence of external ear openings (vs absent); from *S. tritaeniatus* (Bourret) by having a smaller size (SVL 38.5 mm vs 47 mm), fewer midbody scale rows (28 vs 38), and fewer paravertebral scales (61 vs 81); from *S. valentinae* Bragin, Geissler, Trofimets, Neang, Le, Nguyen & Poyarkov by having fewer anterior temporals (2 vs 3), more infralabials (6 vs 4), more midbody scale rows (28 vs 18), more lamellae under finger IV (7 vs 5), more lamellae under toe IV (12 or 13 vs 6), and the presence of external ear openings (vs absent); from *S. veunsaiensis* (Geissler, Hartmann & Thy) by having a larger size (SVL 38.5 mm vs 33.6–35.2 mm), more midbody scale rows (28 vs 20–22), more paravertebral scales (61 vs 51–53), more lamellae under fourth toe (12 or 13 vs 6), and the presence of external ear openings (vs absent); and from *S. yersini* Nguyen, Nguyen, Nguyen, Orlov & Murphy by having a smaller size (SVL 38.5 mm vs 50–56 mm), more paravertebral scales (61 vs 50–55), more anterior temporal (2 vs 1), fewer midbody scale rows (28 vs 32–34), and fewer lamellae under toe IV (12 or 13 vs 18–20).

*Sphenomorphus puhoatensis* sp. nov. is most similar to *S. tamchucensis* and *S. tonkinensis* in several aspects (i.e., body size, midbody scale rows, number of anterior temporals, lamellae under fourth toe and color pattern). However, the new species can be distinguished from *S. tamchucensis* by having fewer supralabials (6 vs 7), fewer lamellae under finger IV (7 vs 8–10), and the presence of enlarged nuchals (vs absent). The new species differs from *S. tonkinensis* by having a smaller ratio of FIL/SVL (0.20 vs 0.24–0.26), fewer midbody scale rows (28 vs 32–34), fewer supraciliaries (8 vs 9), fewer paravertebral scales (61 vs 65–72), fewer lamellae under toe IV (12 or 13 vs 15–19), fewer lamellae under finger IV (7 vs 10–11), and fewer dorsal scale rows on back (6 vs 8).

## Discussion

Our discovery brings the species number of *Sphenomorphus* in Vietnam to 18. The new species is currently found only in Pu Hoat Nature Reserve, a protected area established in 2002 in Nghe An Province. The new species is currently known to occupy a small known range with an estimated area of less than 50 km^2^. The site has been experiencing severe habitat degradation primarily due to agricultural and livestock farming activities. We recommend listing the species as Data Deficient based on the IUCN Red List categories and criteria (IUCN 2025). Further research is needed to assess the population size of and anthropogenic threats to this species and to determine its conservation status.

### Key to the species of *Sphenomorphus* from Vietnam

**Table:** 

1	External ear openings absent	**2**
–	External ear openings present	**5**
2	Forelimb with three digits	*** S. tridigitus***
–	Forelimb with four or five digits	**3**
3	Forelimb with four digits	*** S. tetradactylus***
–	Forelimb with five digits	**4**
4	Midbody scale rows 18; lamellae under fourth finger 5	*** S. valentinae***
–	Midbody scale rows 20–22; lamellae under fourth finger 9	*** S. veunsaiensis***
5	Midbody scale rows 38–44	*** S. maculatus***
–	Midbody scale rows 21–38	**6**
6	SVL of adults less than 56 mm	**7**
–	SVL of adults more than 60 mm	**14**
7	Primary temporal 1	**8**
–	Primary temporals 2	**9**
8	TaL/SVL ratio 1.47–1.51; ear opening with 1–3 lobules; flank without light spots	*** S. buenloicus***
–	TaL/SVL ratio 1.81; ear opening without lobules; flank with light spots	*** S. yersini***
9	Midbody scale rows 38; paravertebral scales 81	*** S. tritaeniatus***
–	Midbody scale rows 28–34; paravertebral scales 58–72	**10**
10	Midbody scale rows 28	**11**
–	Midbody scale rows 30–34	**12**
11	Enlarged nuchals absent; lamellae under fourth finger 8–10	*** S. tamchucensis***
–	Enlarged nuchals in one pair; lamellae under fourth finger 7	*** S. puhoatensis***
12	Supralabials 6; supraciliaries 6	*** S. bacboensis***
–	Supralabials 7, supraciliaries 7–9	**13**
13	Midbody scale rows 30; paravertebral scales 61–62; flank without light spots; free margins of eyelids without white edges	*** S. mimicus***
–	Midbody scale rows 32–34; paravertebral scales 65–72; flank with light spots; free margins of eyelids with white edges	*** S. tonkinensis***
14	Tympanum superficial; supraciliaries 7	*** S. cryptotis***
–	Tympanum deeply sunk; supraciliaries 8–10	**15**
15	Dorsum without dark vertebral stripe or dark dots; upper lateral zone without black stripe	*** S. phuquocensis***
–	Dorsum with dark vertebral stripe or dark dots; upper lateral zone with black stripe	**16**
16	Midbody scale rows 21–26	*** S. annamiticus***
–	Midbody scale rows 30–38	**17**
17	Preocular 1; supraoculars 2–3; back of thigh with a patch of enlarged scales	*** S. incognitus***
–	Preoculars 2; supraoculars 4; back of thigh without a patch of enlarged scales	*** S. indicus***

## Supplementary Material

XML Treatment for Sphenomorphus puhoatensis

## Additional information

### Artificial Intelligence (AI) use

The authors accept full responsibility for the content of the manuscript, including the disclosure of any use of AI.

No AI tools were used in the preparation of this manuscript.

### Author ORCIDs

A.V. Pham https://orcid.org/0000-0002-6023-3418

C.T. Pham https://orcid.org/0000-0001-5158-4526

A.V. Ong https://orcid.org/0000-0003-3683-3832

M.D. Le https://orcid.org/0000-0002-2953-2815

T. Ziegler https://orcid.org/0000-0002-4797-609X

T.Q. Nguyen https://orcid.org/0000-0002-6601-0880

### Data availability

All of the data that support the findings of this study are available in the main text or Supplementary Information.

## Supplementary materials

Supplementary material 1 Phylogenetic data Data type xls Explanation note **table SS1**. Uncorrected (“p”) distance matrix showing percentage pairwise genetic divergence (COI) between the new species and closely related species. **table SS2**. Uncorrected (“p”) distance matrix showing percentage pairwise genetic divergence (16S) between the new species and closely related species. https://binary.pensoft.net/file/1625138This dataset is made available under the Open Database License (http://opendatacommons.org/licenses/odbl/1.0/). The Open Database License (ODbL) is a license agreement intended to allow users to freely share, modify, and use this Dataset while maintaining this same freedom for others, provided that the original source and author(s) are credited. Anh Van Pham, Cuong The Pham, An Vinh Ong, Minh Duc Le, Thomas Ziegler, Truong Quang Nguyen

Supplementary material 2 Principal component analysis (PCA) for the *Sphenomorphus* species group Data type csv https://binary.pensoft.net/file/1625139This dataset is made available under the Open Database License (http://opendatacommons.org/licenses/odbl/1.0/). The Open Database License (ODbL) is a license agreement intended to allow users to freely share, modify, and use this Dataset while maintaining this same freedom for others, provided that the original source and author(s) are credited. Anh Van Pham, Cuong The Pham, An Vinh Ong, Minh Duc Le, Thomas Ziegler, Truong Quang Nguyen
